# Editorial: Endocrine Disruption in Light of Dohad: The Challenges of Contaminants of Emerging Concern in Food and Water

**DOI:** 10.3389/fendo.2022.898736

**Published:** 2022-04-28

**Authors:** Marco Aurelio Romano, Anderson Joel Martino-Andrade, Paulo Cezar de Freitas Mathias, Luiz Felipe Barella, Renata Marino Romano

**Affiliations:** ^1^ Department of Medicine, State University of Midwest (UNICENTRO), Guarapuava, Brazil; ^2^ Department of Physiology, Federal University of Paraná (UFPR), Curitiba, Brazil; ^3^ Department of Biotechnology, Genetics and Cell Biology, State University of Maringá (UEM), Maringá, Brazil; ^4^ Molecular Signaling Section, Laboratory of Bioorganic Chemistry, National Institutes of Health-National Institute of Diabetes and Digestive and Kidney Diseases (NIH-NIDDK), Bethesda, MD, United States

**Keywords:** Endocr disrupting chemicals, DOHaD (development origins of health and disease), Endocrine system and disorders, reproductive toxicity, Obesity, alternative methods in toxicity

Endocrine-disrupting chemicals are substances capable of interacting with the endocrine system, mimicking, interfering with, or blocking the activity of endogenous hormones. These effects may have consequences in all organisms due to the high interaction of the endocrine system with the central nervous system and peripheral organs (1). An important characteristic of the endocrine system is the U-shaped dose-response curve, in which minimal amounts of hormones have a larger effect on the activation of hormone receptors than higher levels (2). This may act in favor of EDCs due to their lower levels but also because the chemicals are commonly presented in a free form in the blood, ready to interact with the endocrine system (2).

The U-shaped dose-response curve commonly presented by EDCs is a challenge for the development and interpretation of data from toxicological assays because these safety tests are based on a linear dose-response. This linearity is the basis of the NOAEL (No Observed Adverse Effect Level) and LOAEL (Lowest Observable Adverse Effect Level).

In addition, the phase of life of exposure may have a strong influence on the development of endocrine disruption. Disturbances during embryogenesis, perinatal, and/or peripubertal periods may reprogram the endocrine system with consequences for proper development and healthy adulthood and aging. In this sense, the developmental origin of health and disease (DOHaD) concept is focused on the evaluation of the impact of alterations in the environment during the windows of sensitivity. An important marker of the DOHaD concept was the association between poor nutrition in early life and impairment of glucose metabolism later on: “the thrifty phenotype hypothesis” (3). Since then, concerns about early exposure and later life consequences have also been identified in other physiological systems in response to alterations in diet or exposure to chemical compounds [revised by (4)].

Contaminants of emerging concern are a group of substances, including pharmaceuticals, pesticides, industrial chemicals, surfactants, and personal care products, that are not commonly monitored in the environment but may have the potential to cause toxicity (5). The occurrence of several pharmaceutical compounds in rivers in all regions of the world (6) and the identification of plasticizers in the human placenta (7) and urine of pregnant women (8) are recent examples of the extent of our inefficient methods of waste disposal and elevated use of pharmaceutics during pregnancy. In this sense, this Research Topic aims to explore the association between chemical compounds during windows of sensitivity and the development of diseases in later phases of life or among generations.

Currently, the development of alternative methods for the assessment of toxicity is highly encouraged. In this context, Nozari et al. proposed an alternative experimental model in transgenic SR4G zebrafish to evaluate the stress response after exposure to compounds with environmental impacts, such as bisphenol A, vinclozolin, and fluoxetine. Their findings support the use of SR4G transgenic larvae as an *in vivo* biomonitoring model to screen chemicals for their stress-disrupting potentials. This is important because there is increasing evidence that brief exposures to environmental pollutants modify the stress response and critical coping behaviors for several generations.

Montagnini et al. evaluated the transgenerational effect of triclosan exposure, a commonly used antimicrobial agent incorporated into a variety of personal care products and in toys, textiles, and plastics, and overt the development and reproductive parameters. Alterations in spermatogenesis were observed in the second generation (F2) after parental exposure (F0). Their findings reinforce the relevance of the DOHaD concept and the necessity to regulate exposure to common chemical compounds during important windows of development.

Mohajer et al. transcended the discussion on the basic paradigm of obesogenesis: excess energy consumption and insufficient physical activity. In this review, new variables are incorporated, increasing the complexity involved in the pathogenesis of obesity: exposure to endocrine chemical disruptors, the interference of these compounds with the endocrine system, the gut microbiome, and the physiology of adipose tissue are explored. This theme is extremely relevant in the DOHaD concept since obesogenic compounds have been shown to cause metabolic disturbances later in life that can even pass into multiple future generations postexposure. The rising rates of obesity and related metabolic diseases are demanding increasing attention on chemical screening efforts and worldwide preventative strategies to keep the public and future generations safe.

Finally, Lu et al. integrate the bioinformatics tools of transcriptome-wide association study with chemical-gene-interaction analysis in the evaluation of environmental endocrine disruptors associated with the age at menarche. This multiapproach methodology identified 120 chemicals and 1580 genes significantly correlated with the age at menarche, of which 11 genes were common to the hypothalamus, pituitary, ovary, uterus, and whole blood, expanding the knowledge of genetic and environmental factors related to the onset of female puberty.

## Author Contributions

All authors listed have made a substantial, direct, and intellectual contribution to the work and approved it for publication.

## Funding

RMR, AJMA and PCFM are fellowship recipients from Conselho Nacional de Desenvolvimento Científico e Tecnológico (CNPq − Brazil).

## Conflict of Interest

The authors declare that the research was conducted in the absence of any commercial or financial relationships that could be construed as a potential conflict of interest.

## Publisher’s Note

All claims expressed in this article are solely those of the authors and do not necessarily represent those of their affiliated organizations, or those of the publisher, the editors and the reviewers. Any product that may be evaluated in this article, or claim that may be made by its manufacturer, is not guaranteed or endorsed by the publisher.
